# Engineered exosomes delivering specific tumor-suppressive RNAi attenuate oral cancer progression

**DOI:** 10.1038/s41598-021-85242-1

**Published:** 2021-03-15

**Authors:** Yutaro Kase, Katsuhiro Uzawa, Sho Wagai, Shusaku Yoshimura, Jun-Ichiro Yamamoto, Yuriko Toeda, Megumi Okubo, Keitaro Eizuka, Toshiaki Ando, Takafumi Nobuchi, Kohei Kawasaki, Tomoaki Saito, Manabu Iyoda, Dai Nakashima, Atsushi Kasamatsu, Hideki Tanzawa

**Affiliations:** 1 Department of Oral Science, Graduate School of Medicine, Chiba University, 1-8-1 Inohana, Chuo-ku, Chiba-shi, Chiba, 260-8670 Japan; 2 Department of Dentistry and Oral-Maxillofacial Surgery, Chiba University Hospital, 1-8-1 Inohana, Chuo-ku, Chiba-shi, Chiba, 260-8677 Japan; 3 Division of Dentistry and Oral Surgery, Eastern Chiba Medical Center, 3-6-2 Okayamadai, Togane-shi, Chiba, 283-8686 Japan; 4 Division of Dentistry and Oral Surgery, Japanese Red Cross Narita Hospital, 90-1 Iida, Narita-shi, Chiba, 286-8523 Japan; 5 Division of Dentistry and Oral Surgery, Kimitsu Chuo Hospital, 1010 Sakurai, Kisarazu-shi, Chiba, 292-8535 Japan; 6 Division of Clinical Research, Medical Mycology Research Center, Chiba University, 1-8-1 Inohana, Chuo-ku, Chiba-shi, Chiba, 260-8673 Japan

**Keywords:** Cancer, Molecular biology, Medical research, Oncology

## Abstract

Exosomes are involved in a wide range of biological processes in human cells. Considerable evidence suggests that engineered exosomes (eExosomes) containing therapeutic agents can attenuate the oncogenic activity of human cancer cells. Despite its biomedical relevance, no information has been available for oral squamous cell carcinoma (OSCC), and therefore the development of specific OSCC-targeting eExosomes (octExosomes) is urgently needed. We demonstrated that exosomes from normal fibroblasts transfected with Epstein–Barr Virus Induced-3 (*EBI3*) cDNA were electroporated with siRNA of lymphocyte cytoplasmic protein 1 (*LCP1*), as octExosomes, and a series of experiments were performed to evaluate the loading specificity/effectiveness and their anti-oral cancer cell activities after administration of octExosomes. These experiments revealed that octExosomes were stable, effective for transferring si*LCP1* into OSCC cells and *LCP1* was downregulated in OSCC cells with octExosomes as compared with their counterparts, leading to a significant tumor-suppressive effect in vitro and in vivo. Here we report the development of a new valuable tool for inhibiting tumor cells. By engineering exosomes, si*LCP1* was transferred to specifically suppress oncogenic activity of OSCC cells. Inhibition of other types of human malignant cells merits further study.

## Introduction

Squamous cell carcinoma (SCC) of the head and neck, including oral SCC (OSCC), is one of the most common groups of lethal human cancers^1^. The principle approach for OSCC patients is surgical resection, which may affect the subsequent quality of life. Chemotherapy by platinum-based and/or molecular-targeting agents could be an alternative treatment. However, there is considerable evidence that these therapies lack acceptable specificity and efficacy. The delivery of RNA molecules, such as small interfering RNA (siRNA), microRNA (miRNA), short hairpin RNA (shRNA), and long non-coding RNA (lncRNA) to silence aberrant expression of genes in a cell is a potentially powerful therapeutic strategy for a variety of malignancies^2,3^. This approach can inactivate specific oncogenes and thus inhibit cell growth and/or cell migration. Despite recent advances in delivering such RNAs, targeting specific tissues or cell types while avoiding nonspecific delivery remains challenging. Exosomes belong to a sub-group of extracellular vesicles (EVs). They are secreted by most cells in the human body and they carry a wide range of functional cargoes^4,5^, playing an essential role in intercellular communication through transfer of their genetic contents. Exosomes escape degradation or clearance in the blood by biological barrier permeability, low immunogenicity and low toxicity^6–8^. Thus, they could be promising vehicles to deliver therapeutic RNAs. In murine pancreatic cancer cells, exosomes carrying a specific siRNA have been functionally modified to target oncogenic *KRAS*^9^, suggesting that these modified exosomes have therapeutic potential for malignant tumors whose molecular target has been identified. In this study, we engineered OSCC-targeted exosomes (octExosomes) that express a transmembrane protein (Epstein–Barr Virus Induced-3 [EBI3]) on their membranes, which is abundantly expressed by OSCC cells. We recently found that *LCP1* overexpression was an essential aspect of various oral cancer progressions, therefore the siLCP1 was selected this RNAi experiments^10^. Moreover, the potential therapeutic efficacy of the octExosomes containing therapeutic RNAi was evaluated in vitro and in vivo.

## Materials and methods

### Ethics statement

Human studies were approved a priori by the ethics committee of Chiba University, Japan (approval #680) and were conducted according to the Declaration of Helsinki. All patients provided written informed consent.

### Cells and culture conditions

Eleven OSCC-derived cell lines (HSC-2, HSC-3, HSC-3-M3, HSC-4, Sa3, Ca9-22, KOSC-2, SAS, Ho-1-u-1, Ho-1-N-1 and SAS-H1), NB1RGB cells (human skin-derived fibroblasts), and HaCaT cells (human keratinocytes) were obtained from the RIKEN BioResource Center (Tsukuba, Ibaraki, Japan), the Japanese Collection of Research Bioresources Cell Bank (Ibaraki, Osaka, Japan) and the Cell Line Service (DKFZ, Heidelberg University, Germany), respectively. They were cultured in Dulbecco’s modified Eagle medium (DMEM) (Sigma-Aldrich, St. Louis, MO, USA) supplemented with 10% fetal bovine serum (FBS) (Sigma-Aldrich) and 50 units/mL penicillin and streptomycin (Sigma-Aldrich) as described^11^. Human normal oral keratinocytes (HNOKs), which were established from healthy oral epithelium specimens collected at Chiba University Hospital, were cultured in oral keratinocyte medium (ScienCell Research Laboratories, Carlsbad, CA, USA) as we described previously^12^.

### Microarray analysis

To screen the mRNA expression profiles of OSCC cells, we used a SurePrint G3 Human GE 8 × 60 K v2 microarray (Agilent Technologies, Santa Clara, CA, USA). We thus compared OSCC-derived cell lines (HSC-2, HSC-3, HSC-4 and Sa3) with HNOKs.

The expression intensity values of significantly differentially expressed genes were obtained based upon a fold-change cutoff greater than 2.0 or less than 0.5 and visualized by volcano plots. A fold-change greater than 4.0 was considered for further validation and analysis. Hierarchical clustering was then conducted using the genes that were shown to be expressed on the OSCC cell membrane. The genes were considered to be associated significantly with the z-score at a false discovery rate exceeding a two-fold change.

### Exosome isolation

For exosome isolation, NB1RGB cells were incubated in Dulbecco’s Modified Eagle’s medium without 10% fetal bovine serum (FBS) (Sigma-Aldrich) for 48 h. Culture supernatants were centrifuged at 2000 × *g* for 20 min to eliminate cells and debris, after which they were filtered through 0.22-μm membranes. The collected samples were then ultracentrifuged at 100,000 × *g* for 1 h at 4 °C to pellet exosomes. The exosome pellets were washed twice in a large volume of PBS and were recovered by centrifugation at 100,000 × *g* for 1 h. Exosomal protein was measured by the Bradford assay with the Bio-Rad Protein Assay Reagent (Bio-Rad Laboratories, Hercules, California, USA) and stored at − 80 °C.

### mRNA isolation and quantitative real-time PCR (qPCR)

We performed qRT-PCR as described previously^13^. For lymphocyte cytoplasmic protein 1 (*LCP1*), recently found as a regulator of OSCC progression in our previous study^10^, we used 5′-AACCCTCGAGTCAATCATTTG-3′ (forward primer) and 5′-TTTGATCTTTTCATAGAGCTGGAA-3′ (reverse primer). For the Epstein-Barr virus Induced-3 (*EBI3*) gene, we used 5′-GAAGTACTGGATCCGTTACAAGC-3′ (forward primer) and 5′-GGAGGACGTGGCTTCAATG-3′ (reverse primer). For the housekeeping gene glyceraldehyde-3-phosphate dehydrogenase (*GAPDH*), we used 5′-AACATCATCCCTGCCTCTACTGG-3′ (forward primer) and 5′-TTGAAGTCAGAGGAGACCACTG-3′ (reverse primer).

### Western blot analysis

Western blot analysis was conducted as described previously^13^. The primary antibodies used in the experiments were as follows: mouse anti-EBI3, # sc-515323 (Santa Cruz Biotechnology, Dallas, TX, USA), 1:200; rabbit anti-CD9 # 13174 (Cell Signaling Technology, Beverly, MA, USA), 1:1000; rabbit anti-CD63 # EXOAB-CD63A-1 (System Biosciences, San Francisco, CA, USA), 1:1000; rabbit anti-CD81 # EXOAB-CD81A-1 (System Biosciences), 1:1000; rabbit anti-LCP1 #HPA019493 (Atlas Antibodies, Stockholm, Sweden), 1:200; mouse anti-α tubulin # sc-5286 (Santa Cruz Biotechnology), 1:200; and, rabbit anti-GAPDH # GTX100118 (Gene Tex, Irvine, CA, USA), 1:1000.

### Engineered cells

NB1RGB cells (human skin-derived fibroblasts) were transfected with an *EBI3* vector (VectorBuilder, Chicago, IL, USA) designed to overexpress human *EBI3* cDNA (oeEBI3 cells) or non-target vector (mock cells) #TR30012 (OriGene Technologies, Rockville, MD, USA) using a Neon electroporator (Life Technologies, Carlsbad, CA, USA). Western blot analysis was utilized to confirm the expression of these proteins in the cells as mentioned above.

### Transmission electron microscopy (TEM)

The TEM analysis was performed as described previously^14^. In brief, exosomes in PBS were placed on the carbon film grid, and they were partially dried. Next, a staining solution of 2% uranyl acetate in water was added to grids for 2 min and the excess liquid was blotted off with filter paper. The grids were dried overnight at room temperature. Grids were analyzed through the use of a HITACHI H-7600 transmission electron microscope (TEM, Hitachi High-Technologies Corporation, Tokyo, Japan) at Hanaichi UltraStructure Research in Japan.

### Nanoparticle tracking analysis (NTA)

The NTA was performed as described previously^15,16^. In brief, the size distribution of the exosomes was analyzed using a Nano Sight LM10 instrument (Marvern instruments, Worcestershire, UK) equipped with NTA software, version 2.3. The particle suspensions were diluted with PBS to a concentration of 10^8^ to 10^9^ particles/mL for analysis.

### Visualization of exosomes

We visualized exosomes as reported previously^17^. In brief, SAS, HaCaT cells and NB1RGB cells were seeded in 8–well chamber slides at a density of 2 × 10^4^ cells/well. After 24 h, the slides were washed twice in D-PBS ( −), and Endothelial Cell Media 2 containing exosomes (5 ng/µL, 10 ng/µL and 20 ng/µL) derived from NB1RGB cells (NB1RGB exo) or from oeEBI3 NB1RGB cells (EBI3 exo) stained by SYTO RNA Select (Thermo Fisher Scientific, Waltham, MA, USA) was added into each well. The exosome-treated cells were cultured for 1 h, 3 h and 6 h at 37 °C under a 5% CO_2_ humidified atmosphere. Then, they were treated with 4% paraformaldehyde solution at room temperature for 20 min. After staining of the nuclei using the ProLong Gold Antifade Reagent with 4′,6–diamidino-2-phenylindole (DAPI; Thermo Fisher Scientific), coverslips were added and the cells visualized under a confocal laser scanning microscope (LSM710; Carl Zeiss, Oberkochen, Germany) and analyzed by FluoView Software (Olympus Optical, Tokyo, Japan).

### Analysis of LCP1 gene expression in tumor samples, its clinical significance and preparation of siLCP1-loaded EBI3 exos (octExosomes)

We recently showed that *LCP1* is one of the essential components for OSCC progression^12^. To further identify the role of *LCP1* in head and neck SCCs (HNSCC), including OSCCs, The Cancer Genome Atlas (TCGA) network cohort (n = 522) was assessed as we described previously^18^. First, we standardized loading conditions of exosomes to achieve a satisfactory and repeatable outcome. Thus, the loading conditions for electroporation were optimized for a Neon electroporation system. The *EBI3* exos were mixed with the Neon electroporation buffer at a 1:1 ratio, and si*LCP1* #sc-43208 (Santa Cruz Biotechnology) or siControl # sc37007 (Santa Cruz Biotechnology) was added to the mixture at a final ratio of siRNA : exosomes protein of 100 pmol:1 μg/mL. Electroporation was then performed at various voltages per the manufacturer's instructions with the pulse width set at 10 ms. The effect of pulse time was also assessed at the optimized voltage. After electroporation, one unit of RNase A was added to the mixture to eliminate free si*LCP1* outside the exosomes. To reduce undesirable electroporation-induced si*LCP1* precipitation during the loading process, EDTA was added.

### Proliferation/migration/invasion assays

SAS and HSC-3 were placed in 6-well plates at 1 × 10^4^ cells/well in proliferation assays. The si*LCP1*-loaded EBI3 exos (octExosomes) or siControl-loaded EBI3 exos (siControl exosomes) were added at doses of 10 µg/mL for 120 h. Cells were counted every 24 h. The cell lines were assessed for viability using Luna Automated Cell Counter (Logos Biosystems, Annandale, VA, USA). All experiments were performed in triplicate.

For live-cell imaging, the cells were seeded in 6-well plates with 10% FBS/DMEM until a confluent monolayer formed. Using a micropipette tip, one wound was created in the middle of each plate. We incubated plates at 37 °C at 5% carbon dioxide with free-serum medium, and live cell migration was captured after 12 and 24 h. The wound area that was free of cells was calculated using Lenaraf220b software (http://www.vector.co.jp/soft/dl/win95/art/se312811.html).

Matrigel invasion assays were carried out using Matrigel-coated Transwell inserts (8 μm pores) (Becton–Dickinson, Franklin Lakes, NJ, USA) following the manufacturer's instructions. Two mL of DMEM with 10% FBS was placed in the lower wells. Proliferating si*LCP1* cells or siControl cells (2.0 × 10^5^ cells per mL) were loaded into each of the upper wells and monitored after 72 h for the migrating cells.

### In vivo targeting and biodistribution in a tumor xenograft mouse model by IVIS imaging

Animal handling and all animal experiments followed the ARRIVE guidelines. BALB/C-nu mice were used to study the in vivo targeting and biodistribution of the octExosomes based on the previous report^19^. In brief, female BALB/cAnNCrj-nu/nu mice (Oriental Yeast Co., Ltd., Andover, MA) were engrafted subcutaneously with 1 × 10^7^ SAS or HSC-3 cells on the back, and the tumor volume was calculated as (width)^2^ × (length)/2. After the injection, the mice with a palpable tumor > 100 mm^3^ in size were chosen for this study. For imaging of fluorescently labeled exosomes, a stock solution of the lipophilic near-infrared dye XenoLight DiR (Caliper Life Sciences, Hopkinton, MA, USA) was prepared in ethanol. The octExosomes were incubated with 2 µmol/L DiR for 30 min, washed with 10 mL of PBS, and then injected intravenously through the tail vein at a dosage of 1.5 mg/kg. Six h after injection and every 3 days, Dir fluorescence in the tumor-bearing mice was captured with a Xenogen IVIS-200 optical in vivo Imaging system (Caliper Life Sciences). The tumors were measured every 3 days. The weight of mice was measured every 3 days during the experiment. No obvious decrease in the body weight of any mouse was detected after drug treatment. Upon completion of treatment (18 days), tumor grafts were harvested. All experimental procedures were approved by The Institutional Animal Care and Use Committee of the Chiba University (approval number, 1-126).

### Immunohistochemistry

For histological analysis, paraffin-embedded samples were first deparaffinized and rehydrated based on standard protocols as we described previously^13^. In brief, the blocking of non-specific antigens and endogenous peroxidase activity was carried out with a peroxidase blocking reagent (serum and 3% hydrogen peroxide), followed by overnight incubation with anti-LCP1 #HPA019493 (Atlas Antibodies), 1:200 and anti-PCNA #GTX100539 (Gene Tex), 1:500 at 4 °C. A ChemMate DAKO EnVision Detection Kit (Peroxidase/DAB, Rabbit/Mouse; DakoCytomation, Glostrup, Denmark) was used to immunostain the slides in accordance to the manufacturer’s protocols.

### Statistical analyses

All data are expressed as the mean (±) standard deviation. Statistical analysis was conducted using Student’s t test. *P* < 0.05 was considered to indicate statistical significance.

## Results

### Development of octExsomes

**Figure 1:**
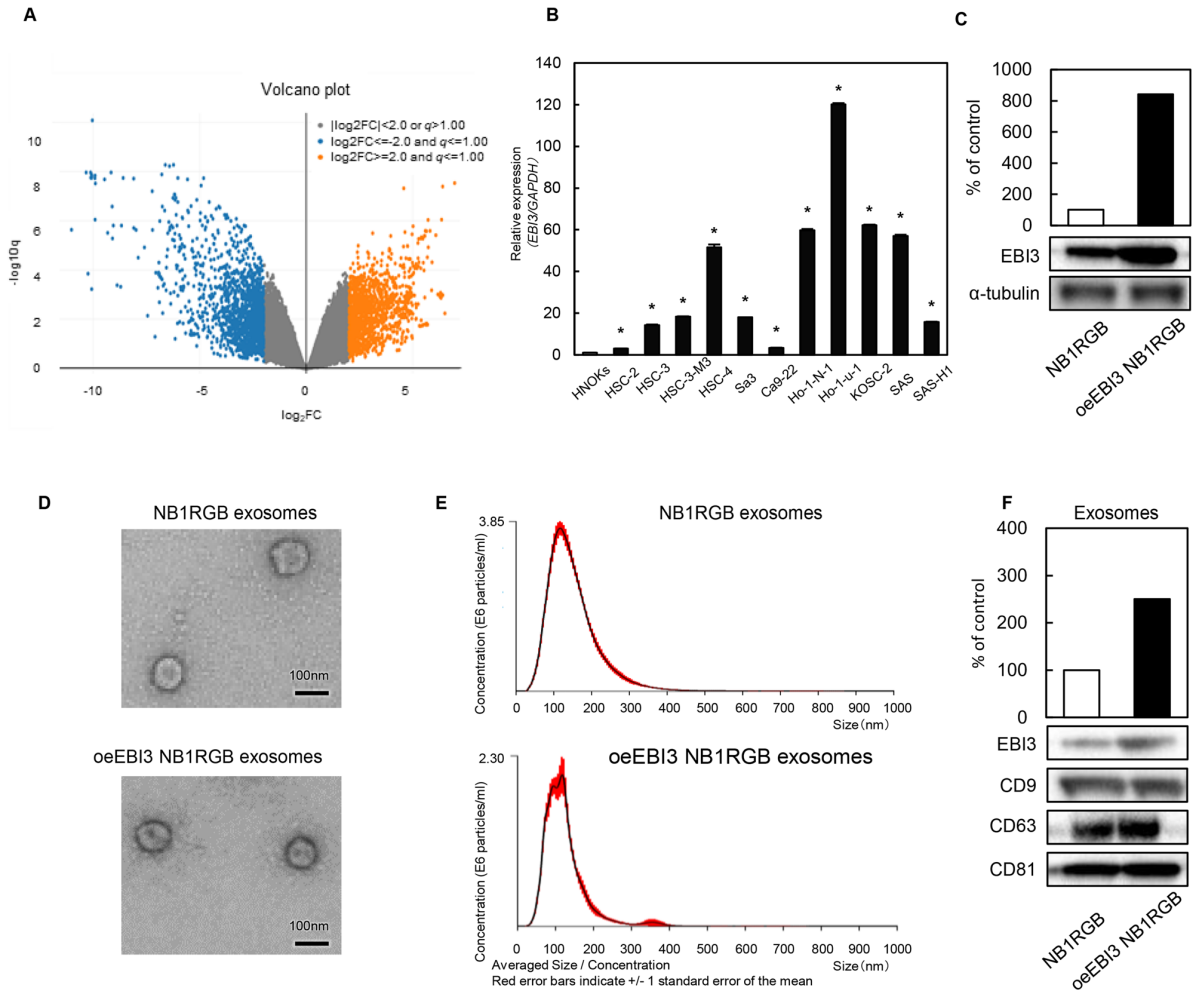
Expression of EBI3 in OSCC, normal cells and exosomes. (**A**) Volcano plots of significant genes with *P* < 0.01 and more than a two-fold change or less than a 0.5-fold change expressed in 1536 upregulated genes and 3381 downregulated genes. The x-coordinate represents the (log_2_) fold-change (FC) and y represents the t-statistic or − log_10_q of the *P* value. (**B**) A qPCR analysis of *EBI3* transcripts in HNOKs and OSCC cell lines. The levels of *EBI3* transcripts were normalized to the expression of *GAPDH*. Data are expressed as ratios of *EBI3* to *GAPDH*. Data are presented as means ± SD (n = 3). *P < 0.05. (**C**) Increased EBI3 in transfected NB1RGB cells was evident when compared to the mock cells. Densitometric EBI3 protein data were normalized to α-tubulin protein levels. (**D**) Electron micrograph of NB1RGB exosomes and oeEBI3 NB1RGB exosomes. (**E**) Size distribution of NB1RGB exosomes and oeEBI3 NB1RGB exosomes measured by NTA peaking at 100 nm diameter. (**F**) EBI3 Western blots of the corresponding exosomes. Note that EBI3-transfected NB1RGB cells released greater amounts of EBI3-expressing exosomes than control cells. CD9, CD63 and CD81 expressions were analyzed as an exosomal markers.

To direct the targeting of exosomes, they were engineered to express peptides that could recognize sequences abundantly present on OSCC cell membranes. We performed gene expression analyses of microarray data collected from OSCC cell lines and HNOKs. Volcano plots show that 1536 upregulated genes and 3381 downregulated genes were significantly changed more than two-fold in their z-scores (Fig. 1A). Among the significantly upregulated genes, unsupervised hierarchical clustering of all the differentially expressed genes (n = 344) showed clear OSCC cell membrane gene expression patterns. Of them, 6 were confirmed to be commonly upregulated in the expression profiles of 4 of the OSCC cell lines. The *EBI3* gene was the only one to increase its mRNA expression level in all OSCC-derived cell lines that we examined (Fig. 1B). Thus, it was selected for further studies in vitro and in vivo. Expression of EBI3 protein was confirmed by Western blot analysis of transfected NB1RGB cells (Fig. 1C). These modifications did not appear to affect the physical properties (size and shape) of the engineered exosomes (eExosomes) and exosomes released from untransfected NB1RGB cells (NB1RGB exosomes) based on electron microscopy (Fig. 1D) and NTA (Fig. 1E). EBI3 protein was significantly upregulated in EBI3-transfected NB1RGB cells and was incorporated into the NB1RGB cell–derived exosomes according to Western blots (Fig. 1F).

**Figure 2:**
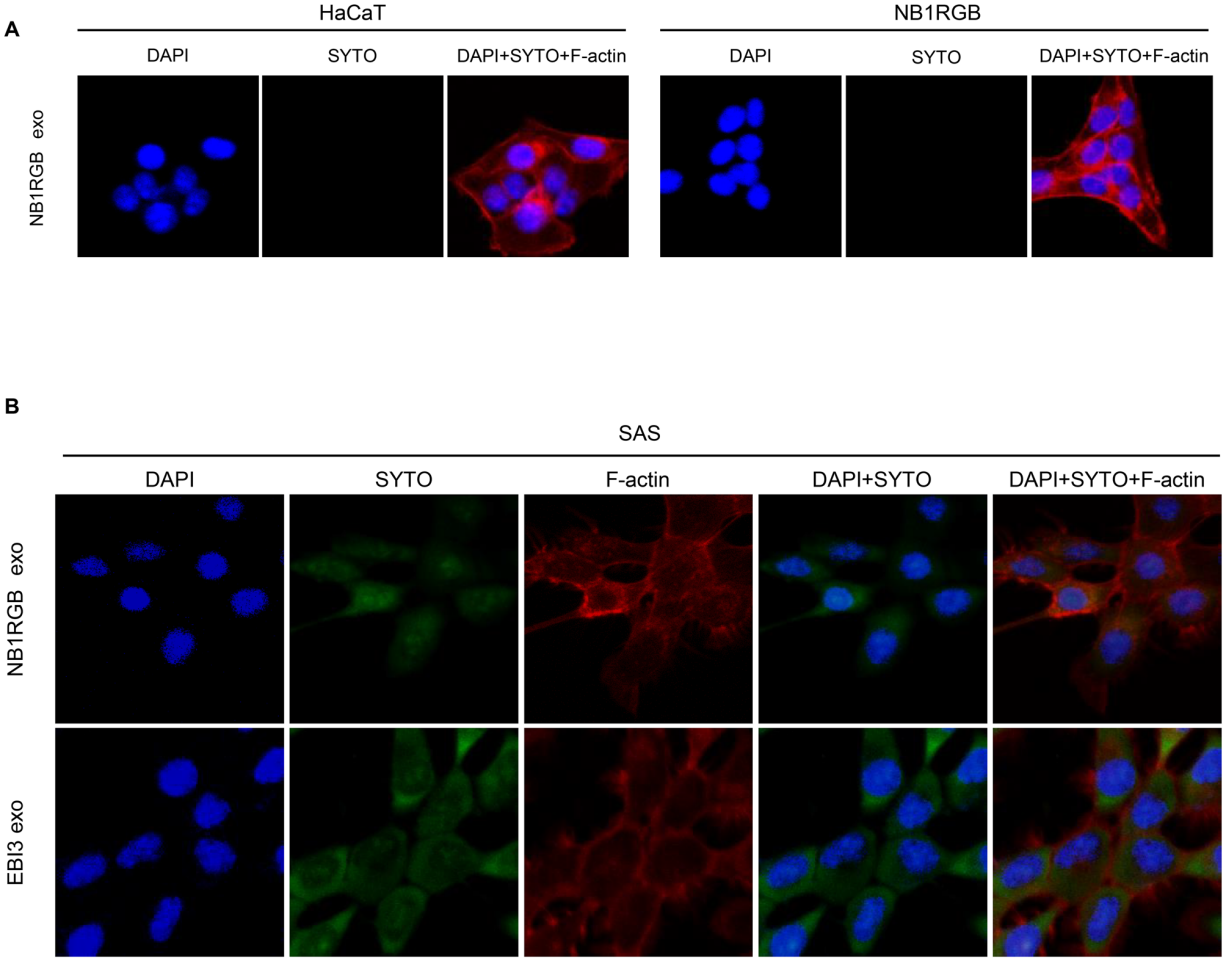
Uptake of visualized exosomes under a confocal laser scanning microscope. (**A**) Exosomes from NB1RGB cells were stained with the SYTO RNA Select Reagent (SYTO). HaCaT cells and NB1RGB cells were treated with exosomes derived from NB1RGB cells (NB1RGB exo). Note that immuno-reaction for SYTO (in green) was not evident in these cells. (**B**) NB1RGB exo or EBI3 exo were stained with SYTO. Note that accumulated SYTO staining was detected in EBI3 exo.

Stained exosomes (NB1RGB exos and EBI3 exos) (5 ng/µL, 10 ng/µL and 20 ng/µL) were added to the culture media of SAS, HaCaT cells and NB1RGB cells. Uptake was examined by confocal laser scanning microscopy. Green fluorescence was not detected in HaCaT cells and NB1RGB cells 6 h after addition of SYTO RNA Select-stained NB1RGB exos (Fig. 2A). In addition, 5 ng/µL, 10 ng/µL and 20 ng/µL stained NB1RGB exos or EBI3 exos were added to the culture media of SAS and uptake was examined as above. Green fluorescence was detected in SAS 1 h, 3 h and 6 h after addition of SYTO RNA Select-stained EBI3 exos. We found that the optimal conditions were 10 ng/µL and 3 h (Fig. 2B, Supplementary Fig. 4). However, the detailed mechanisms of their cellular uptake are still unknown. Some recent reports suggested that endocytosis has been reported to be a major pathway for the cellular uptake of exosomes^20,21^. Therefore, these results suggested that exosomes released from oeEBI3 NB1RGB cells were more taken up in SAS, and it may be speculated via endocytosis.

Regarding the bioinformatics analysis of *LCP1*, although *LCP1* mRNA expression status was not associated with lower overall survival (OS), its overexpression was linked to lower OS among the patients with metastatic regions (Supplementary Fig. 1A,B). Those findings suggested that si*LCP1* was a good candidate for an eExosome cargo for targeting OSCC cells.

### The octExosomes suppress the progression of oral cancer cells in vitro

**Figure 3:**
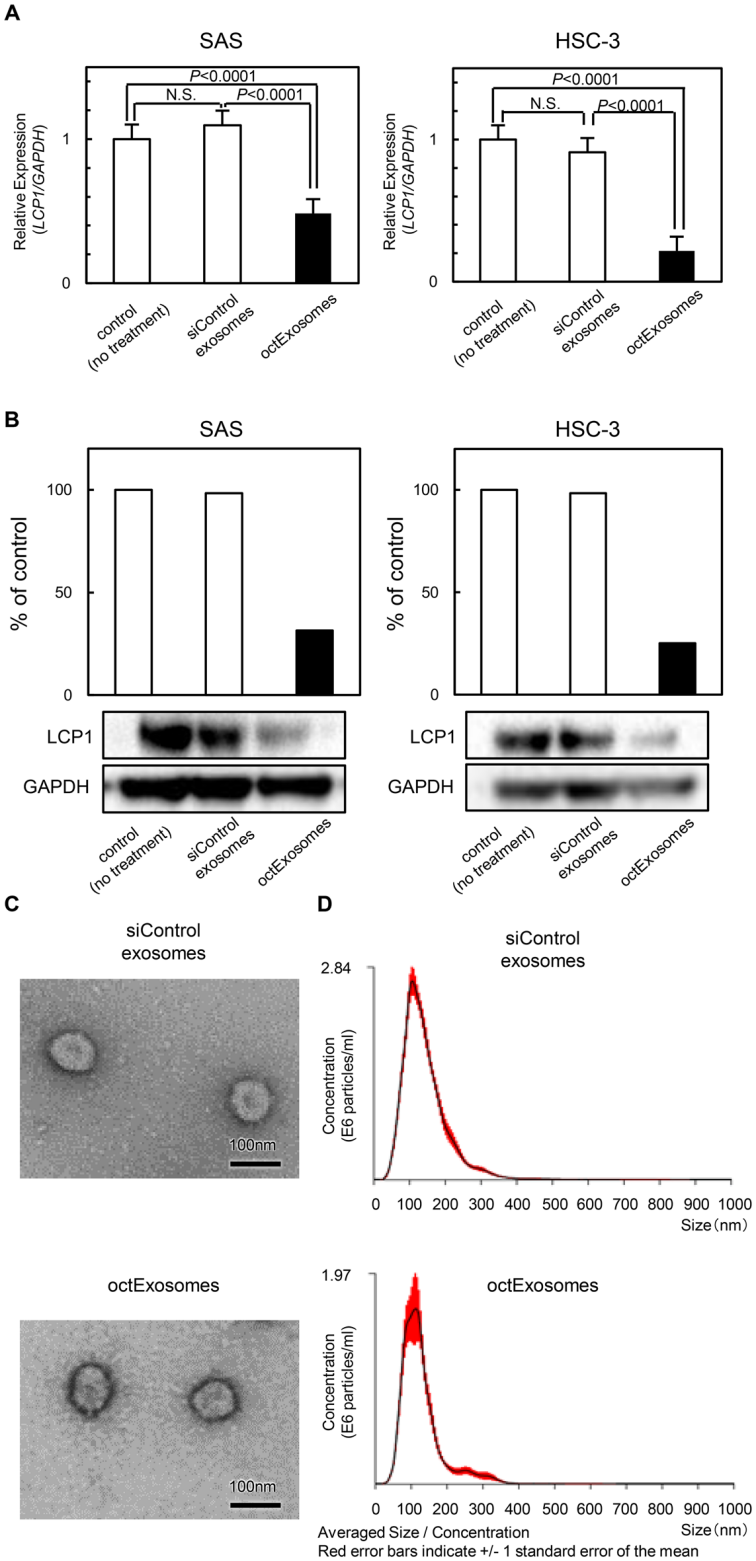
Influence of si*LCP1* on OSCC cells treated with octExosomes or siControl exosomes. (**A**) Significant downregulation of *LCP1* mRNA and (**B**) LCP1 protein levels, indicating high delivery efficiency of si*LCP1* was achieved. (in **A**, data are presented as means ± SD, n = 3. *P < 0.05) (**C**) Electron micrograph of siControl exosomes and octExosomes. (**D**) Size distribution of siControl exosomes and octExosomes as measured by NTA peaking at 100 nm diameter. These modifications did not alter the physical properties of the modified exosomes.

**Figure 4:**
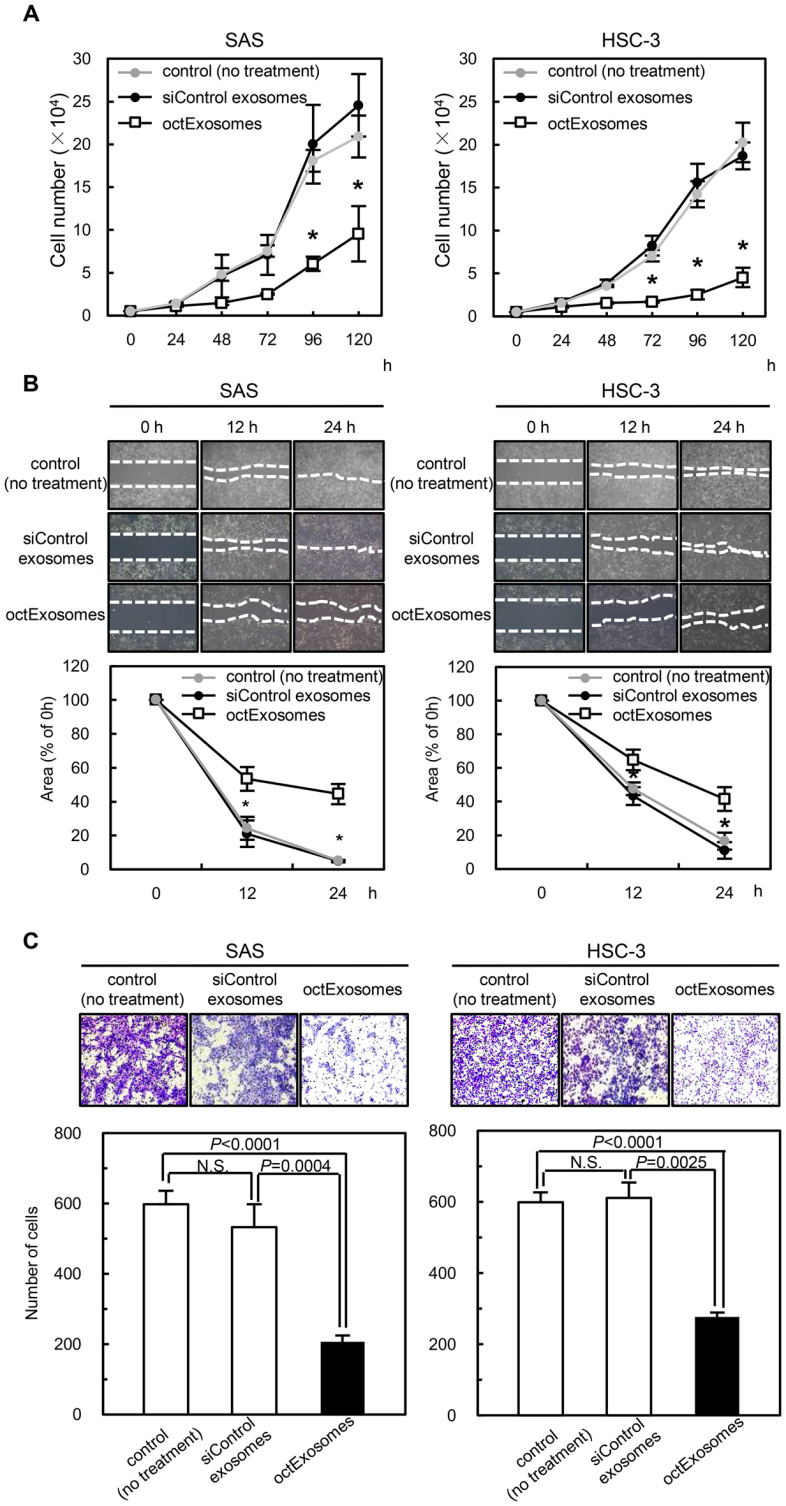
Tumor progression assay of OSCC cells transfected with octExosomes. (**A**) Proliferation assays for SAS and HSC-3 cells treated with octExosomes or siControl exosomes. (**B**) Migration assays for SAS and HSC-3 cells transfected with octExosomes or siControl exosomes. (**C**) Invasion assays for SAS and HSC-3 cells transfected with octExosomes or siControl exosomes. (Data are presented as mean ± SD. N = 3. *P < 0.05). Note that the silencing of *LCP1* drastically reduced the proliferation, migratory and invasive abilities of SAS and HSC-3.

To assess whether the si*LCP1*-loaded EBI3 exos (octExosomes) would be able to specifically deliver si*LCP1 *in vitro, we performed qPCR and Western blot analyses. As expected, we detected significant downregulation of *LCP1* in octExosome-treated cell lines (Fig. 3A,B). Adding siControl exosomes to the cells did not result in *LCP1*-inhibition (Fig. 3A,B), suggesting high efficacy of exosome-mediated delivery of si*LCP1* by octExosomes. Furthermore, these modifications did not appear to affect the physical properties (size and shape) of the octExosomes and siControl exosomes based on electron microscopy (Fig. 3C) and NTA (Fig. 3D). We then examined whether octExsosomes interrupted cell growth/migration/invasion in vitro. Significant cell growth inhibition was apparent in OSCC cells treated with octExosomes, while no growth inhibition of cells treated with siControl exosomes was observed (Fig. 4A). In addition, the silencing of *LCP1* expression by octExosomes drastically weakened the cells’ migratory and invasive abilities, but not in the siControl exosome group. (Fig. 4B,C).

### The octExosomes suppress the progression of oral cancer cells in vivo

**Figure 5:**
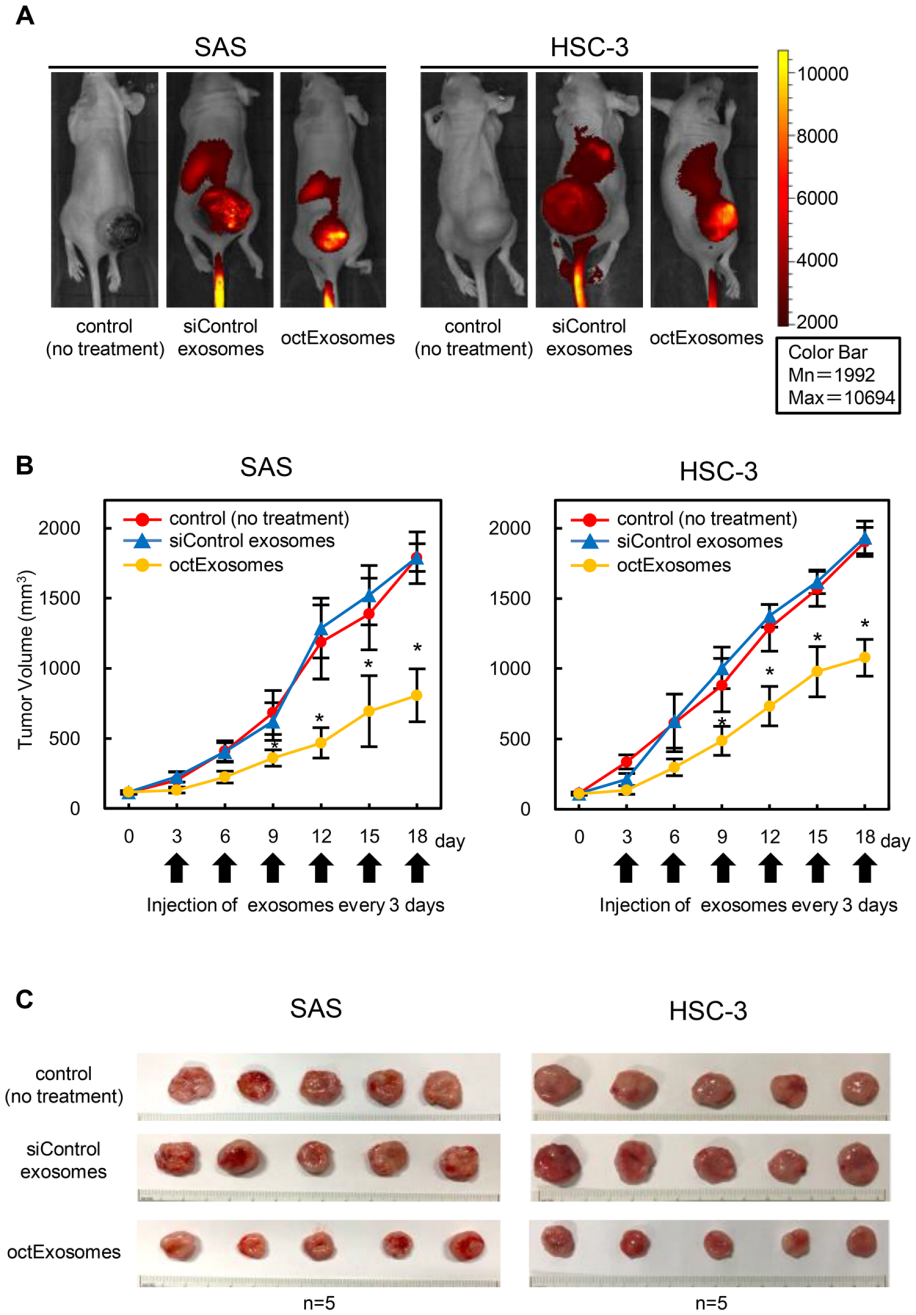
In vivo fluorescent images of SAS and HSC-3 tumor-bearing mice after i.v. injections of exosomes. (**A**) Fluorescent images of whole-body imaging of SAS and HSC-3 tumor-bearing mice after treatment with exosomes. Strong accumulation of fluorescence was observed by whole-mouse imaging in the tumor area. (**B**) Tumor volume growth curves. (Arrows, i.v. injections of exosomes; data are presented as mean ± SD. N = 5. *P < 0.05). (**C**) Pictures of local tumors from mice bearing SAS cells or HSC-3 cells. Based upon the series of transplantations, we confirmed that tumor growth was suppressed at considerably higher levels in the octExosome group than in siControl exosome and control groups.

**Figure 6:**
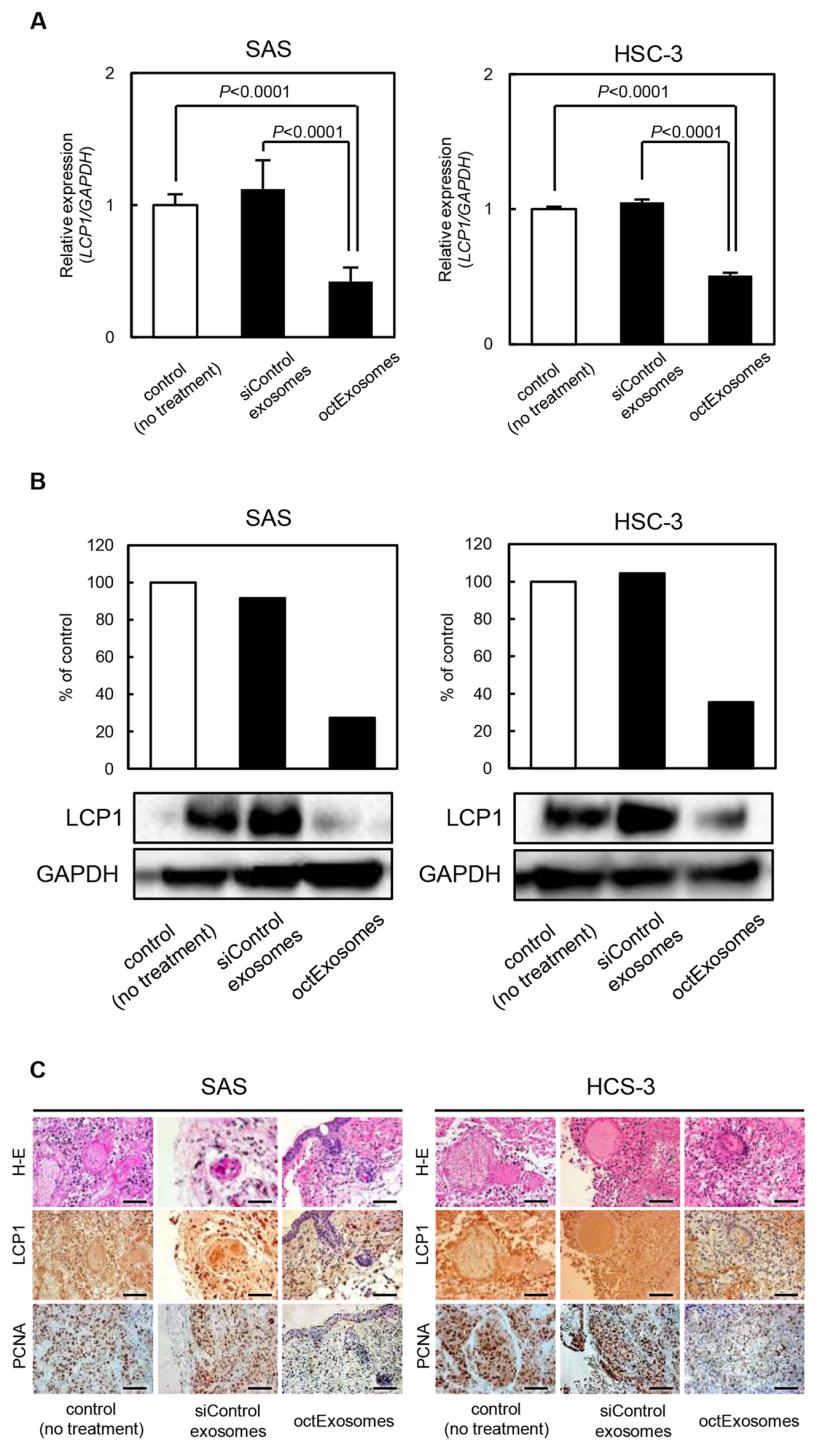
Tumor xenograft studies after i.v. injections of exosomes. (**A**) Quantitation of *LCP1* mRNA change. (Data are presented as means ± SD. N = 5. *P < 0.05.) (**B**) Quantitation of LCP1 protein change. **A, B.** Based on qPCR and Western blot analyses. Note that LCP1 was considerably suppressed in the octExosome group compared to siControl exosome and control groups at both the mRNA level and protein level. (**C**) Immunohistochemical analysis of tumor LCP1 and PCNA changes. LCP1 and PCNA expressions were considerably suppressed in the octExosome group. Scale bars indicate 50 μm.

We next evaluated the tumor targeting effect of octExosomes in vivo. As shown in Fig. 5A, a strong accumulation of Dir fluorescence was observed by whole-mouse imaging in the tumor area for the siControl exosome and octExosome group. It is noteworthy that the color intensity for exosome accumulation was much higher in the tumor area (Fig. 5A). Furthermore, while siControl exosomes did not suppress tumor growth, adding the octExosomes significantly inhibited tumor growth. The sizes of tumors at day 18 were ranked as follows: octExosomes < siControl exosomes ≈ control, Fig. 5B, C. No significant body weight loss was observed across different groups during the experimental period (Supplementary Fig. 2). Among the xenografted tumors, both *LCP1* mRNA levels and LCP1 protein levels were significantly reduced in octExosomes-tumors compared to the siControl exosome group (Fig. 6A, B). Immunohistochemical analyses showed clear differences in LCP1 and PCNA expression status between the two groups (octExosomes versus siControl exosomes; Fig. 6C). PCNA, a marker of cell proliferation, level was considerably suppressed in the octExosome group. Therefore, we suggest that octExosomes treatment led to markedly suppression of tumor growth through downregulation of LCP1.

## Discussion

Accumulating evidence has suggested that exosomes released from human cells could be ideal nanocarriers of therapeutic agents for clinical use^22–24^. However, it is not clear how best to endow exosomes with the efficiency to specifically deliver therapeutic molecules to target cells. In addition, cancer-derived exosomes will regulate/facilitate organ-specific metastases^25^, indicating that the development of specific cancer-targeting exosomes from non-cancerous cells is required. Here, we report that the progression of oral cancer cells was inhibited by treatment with specific exosomes (octExosomes). They were constructed by modifying normal fibroblast-derived exosomes such that they expressed transmembrane EBI3 on the surface and enclosed si*LCP1*.

We identified gene expression signatures common to OSCC transmembrane proteins and identified a commonly-overexpressed gene in OSCC cell lines (Fig. 1B), *EBI3* (Fig. 1A, B). *EBI3* regulates the immune system and multiple cytokines^26^. In addition, EBI3-deficient mice show prominent abnormalities in the glomerular basement membrane^27^. Moreover, *EBI3*-deficiency leads to downregulated expression of the adhesion molecule VCAM1^28^. A recent report suggested that IL-35, a subunit of which is EBI3 protein, promotes metastasis of human pancreatic cancer^29^ by inducing the endothelial adhesion molecule, ICAM1. Further, *EBI3* is frequently expressed in a subset of human malignancies^30–32^, suggesting that EBI3 may play a functional role in cell–cell adhesion during the progression of human cancers.

The interaction between transmembrane EBI3 and octExosomes in the cancer microenvironment is still unknown. Since EBI3 also constitutes homodimeric structures^33,34^, we speculated here that our octExosomes directly recognized EBI3 protein produced from the OSCC cells. Thus, further studies on cellular uptake and membrane fusion mechanisms in exosomes are necessary. Nonetheless, this molecule could be a candidate surface component of OSCC-specific exosomes.

Most human cells release exosomes^35^. However, due to the differences in their contents and their surface compositions, it is crucial to consider their characteristics with regard to therapy. From a clinical standpoint, the above-mentioned characteristics probably require modification. The contents of exosomes derived from cancer cells may promote cancer progression^36^. Thus, therapeutic exosomes must be derived from normal cells. We chose to modify normal human fibroblast (NB1RGB)-derived exosomes. After transfecting NB1RGB cells with *EBI3*, we observed a robust accumulation of modified exosomes. There was no significant influence on exosomal size (Fig. 1D, E). Thus, we suggest that the modified exosomes from NB1RGB cells could be useful for treating OSCC cells, possessing high efficacy and low oncogenicity on targets.

We recently found that *LCP1* overexpression was an essential aspect of oral cancer progression^10^. The silencing of *LCP1* by siRNA suppressed both cancer cell growth and metastatic phenotypes, and *LCP1*-positive OSCC cases were closely associated with the tumor size and regional metastasis^10^. TCGA cohort analysis indicated a significant relationship between *LCP1* overexpression and lymph node status (Supplementary Fig. 1C, D). Considering the impact of *LCP1* oncogenic activation, we speculated that more concerted efforts were needed to reduce *LCP1* expression. Towards that end, we examined the anti-tumor effects achieved by loading si*LCP1* into the modified exosomes. Electroporation techniques allowed us to load large amounts of si*LCP1* into highly stable exosomes (Fig. 3D). Our production method relies on ultracentrifugation, which could include non-exosomal contaminants. However, the octExosome preparations did not yield any measurable side effects and showed consistent in vitro and in vivo efficacy (Figs. 4 and 5 and Supplementary Fig. 2). In the present study, we found that octExosomes accumulated around OSCC cells and suppressed LCP1 expression in OSCC cells/tumors in vitro and in vivo (Figs. 4, 5, 6). Lower expression of LCP1 protein was observed in xenografted OSCC cells compared to the siControl group, suggesting that si*LCP1* was effectively loaded into cancer cells via octExosomes (Fig. 6B, C).

The field of exosome-based drug delivery has been pursued for a number of years^37^. Here, we demonstrated that specific targeting and anti-tumor effects can be achieved with fibroblast cell exosomes. Our preclinical data provide insights into this novel approach to the treatment of OSCC patients. Future work should focus on modified exosomes delivering tumor-suppressive siRNAs with greater potency or other types of small RNAs. Those studies are needed to advance the understanding of exosome-based treatment for OSCC as well as other types of human malignant tumors.

## Supplementary Information

Supplementary Figures

## Data Availability

These microarray data were deposited in the NCBI Gene Expression Omnibus database under GEO accession number GSE146483. Other datasets generated during and/or analyzed during the current study are available from the corresponding author on reasonable request.
