# Supplementary material for: Engineered exosomes delivering specific tumor-suppressive RNAi attenuate oral cancer progression
Source: Sci Rep. 2021 Mar 15;11:5897. doi: 10.1038/s41598-021-85242-1 (PMC7960743; doi:10.1038/s41598-021-85242-1)
Supplement: Supplementary file 1 — Supplementary Figures [file 41598_2021_85242_MOESM1_ESM.pdf]

Supplementary information for

Engineered exosomes delivering specific tumor-suppressive RNAi attenuate oral cancer progression

Yutaro Kase[1], Katsuhiro Uzawa[*1,2], Shou Wagai[1], Shusaku Yoshimura[1,3], Jun-Ichiro Yamamoto[1,4], Yuriko Toeda[1], Megumi Okubo[1], Keitaro Eizuka[1,5], Toshiaki Ando[1], Takafumi Nobuchi[1], Kohei Kawasaki[1], Tomoaki Saito[2], Manabu Iyoda[2], Dai Nakashima[1], Atsushi Kasamatsu[2], and Hideki Tanzawa[1,6]

[1]*Department of Oral Science, Graduate School of Medicine, Chiba University, 1-8-1 Inohana, Chuo-ku, Chiba-shi, Chiba 260-8670, Japan*
[2]*Department of Dentistry and Oral-Maxillofacial Surgery, Chiba University Hospital, 1-8-1 Inohana, Chuo-ku, Chiba-shi, Chiba 260-8677, Japan*
[3]*Division of Dentistry and Oral Surgery, Eastern Chiba Medical Center, 3-6-2 Okayamadai, Togane-shi, Chiba 283-8686, Japan*
[4]*Division of Dentistry and Oral Surgery, Japanese Red Cross Narita Hospital, 90-1 Iida, Narita-shi, Chiba 286-8523, Japan*
[5]*Division of Dentistry and Oral Surgery, Kimitsu Chuo Hospital, 1010 Sakurai, Kisarazu-shi, Chiba 292-8535, Japan*
[6]*Division of Clinical Research, Medical Mycology Research Center, Chiba University, 1-8-1 Inohana, Chuo-ku, Chiba-shi, Chiba 260-8673, Japan*

[*]Corresponding Author:
Katsuhiro Uzawa, DDS, PhD, FIBCSOMS
Department of Oral Science
Graduate School of Medicine, Chiba University
1-8-1 Inohana, Chuo-ku
Chiba, 260-8670, Japan
Phone: +81-43-226-2300
E-mail: uzawak@faculty.chiba-u.jp

Supplementary Figure S1

The mRNA expression data (level 3) and clinical data (up to January 16, 2016) were obtained from TCGA data portal. The mRNA expression of *LCP1* in HNSCC samples was analyzed with the Illumina HiSeq mRNA Seq platform. All analyses were performed in R. Student's t-test was used to evaluate differences among several clinicopathological factors, including the sample types of normal tissue or cancer tissue, metastatic diagnosis, stage, T status, and N status of HNSCC as stratified by the *LCP1*-associated genomic subtype, and Kaplan–Meier (KM) survival curves of overall survival (OS) plotted for TCGA cohorts. In this study the OS was defined as the day of death or the day of completing follow-up from the day of surgery. Using the KM curve in the R package, survival, the association between the grouping and the actual prognosis information was analyzed. The threshold was set as log-rank p-value < 0.05.
Although *LCP1* expression status is not associated with OS **(A)**, the lower *LCP1* group revealed better OS when compared to the higher group in patients with metastatic lesion **(B)**. Among the OSCC examined, a higher expression of *LCP1* was detected in patients with metastatic lesion compared with the non-metastatic group. The status of *LCP1* expression in the metastatic group (n = 283) and the non-metastatic group (n=239) was based on the Illumina HiSeq mRNA Seq platform. The relative *LCP1* expression level of the metastatic group and the non-metastatic group ranged from 6.59 to 14.02 (median, 11.43) and 7.18 to 13.94 (median, 10.65), respectively **(C)**. The *LCP1* status in the metastatic group was closely associated with lymph node metastasis status of the neck. *LCP1* expression in the lymph nodes in the N0 group (n = 238), N1 group (n = 104), N2 group (n = 165) and N3 group (n = 12). The relative *LCP1* expression level of the N0 group ranged from 7.18 to 13.94 (median, 10.65), N1 group ranged from 7.16 to 13.89 (median, 11.46), N2 group ranged from 6.59 to 14.02 (median, 11.30) and N3 group ranged from 10.18 to 13.60 (median, 11.46). **(D)**.

Supplementary Figure S2

We found that the average body weight of the octExosomes-treated mice never dropped lower than that of the control group at any time points.

Supplementary Figure S3

Full-length blots of Figure 1C & 1F

The samples derive from the same experiment and that gels/blots were processed in parallel.

Full-length blots were captured at the indicated exposure times (1, 3 and 5 seconds). Red dot rectangle, the cropped image for Figure 1C & 1F.

Supplementary Figure S4

Fluorescent immunostaining images of Figure 2B of SYTO and DAPI + SYTO + F-actin
, the intensity was quantified by means of ImageJ.

Supplementary Figure S5

Full-length blots of Figure 3B

The samples derive from the same experiment and that gels/blots were processed in parallel.

Full-length blots were captured at the indicated exposure times (1, 3 and 5 seconds). Red dot rectangle, the cropped image for Figure 3B.

Supplementary Figure S6

Full-length blots of Figure 6B

The samples derive from the same experiment and that gels/blots were processed in parallel.

Full-length blots were captured at the indicated exposure times (1, 3 and 5 seconds). Red dot rectangle, the cropped image for Figure 6B.

Supplementary Figure S1

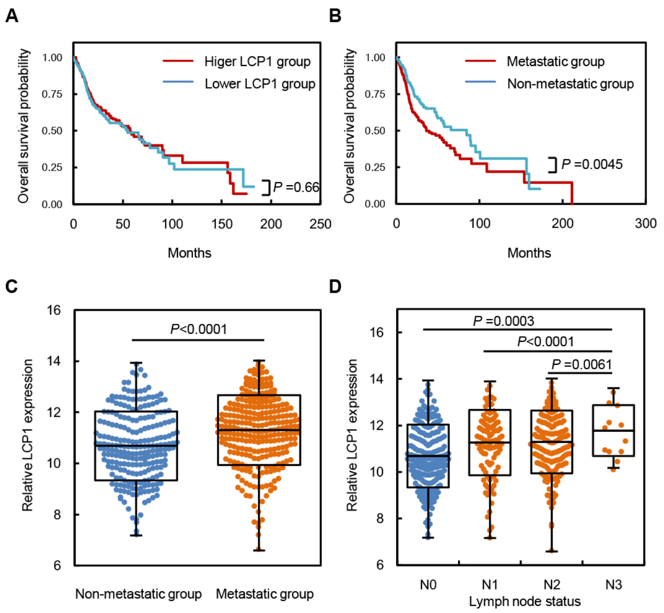

Supplementary Figure S2

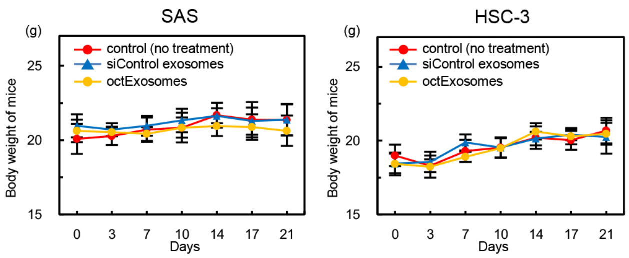

Supplementary Figure S3

(Full length blots of Figure 1C)

(Figure 1C)

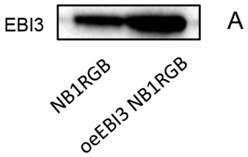

A

EBI3

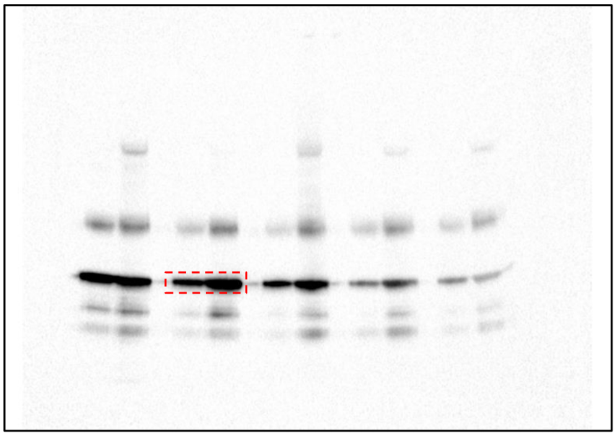

(3 seconds)

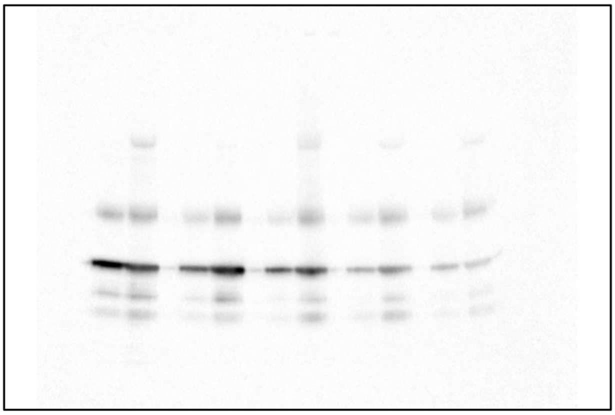

(1 second)

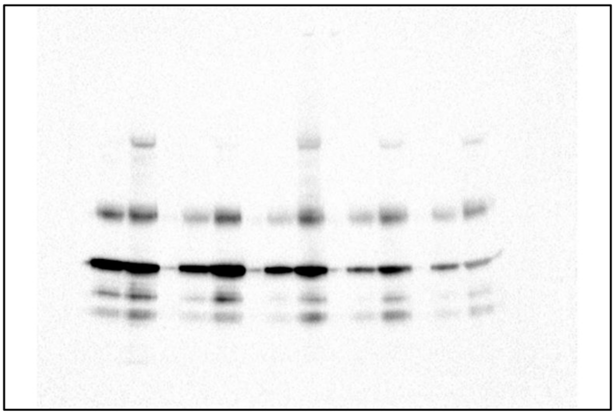

(5 seconds)

Supplementary Figure S3

(Full length blots of Figure 1C)

(Figure 1C)

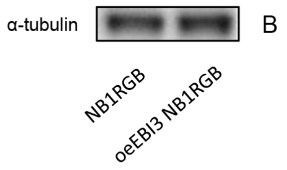

B

α-tubulin

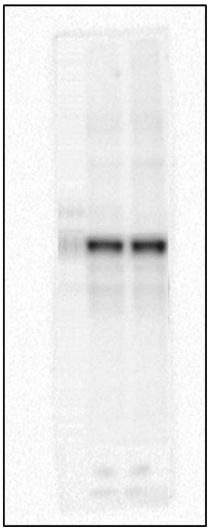

(1 second)

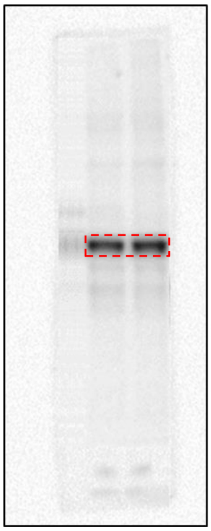

(3 seconds)

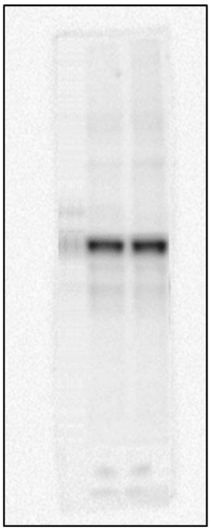

(5 seconds)

Supplementary Figure S3

(Full length blots of Figure 1F)

(Figure 1F)

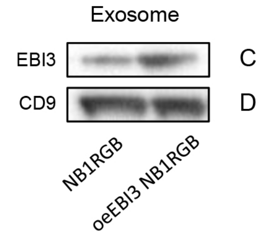

C

EBI3

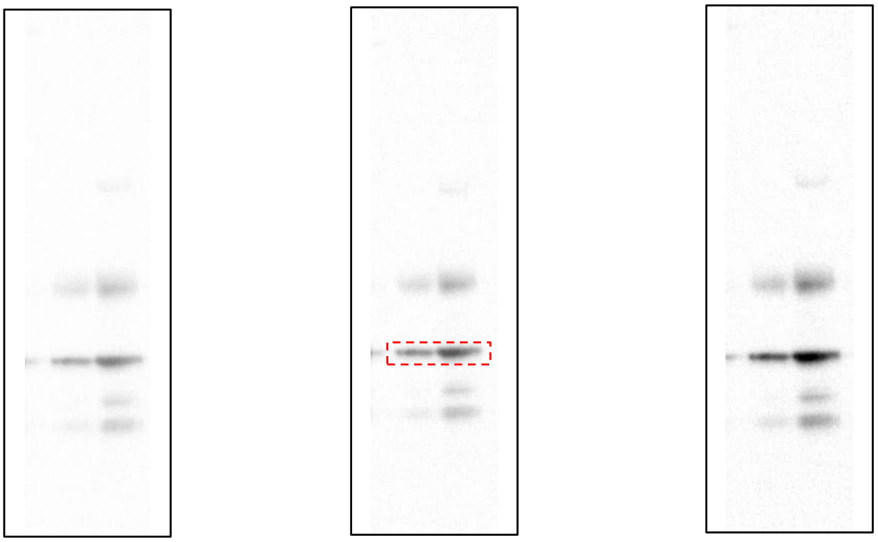

(1 second) (3 seconds) (5 seconds)

D

CD9

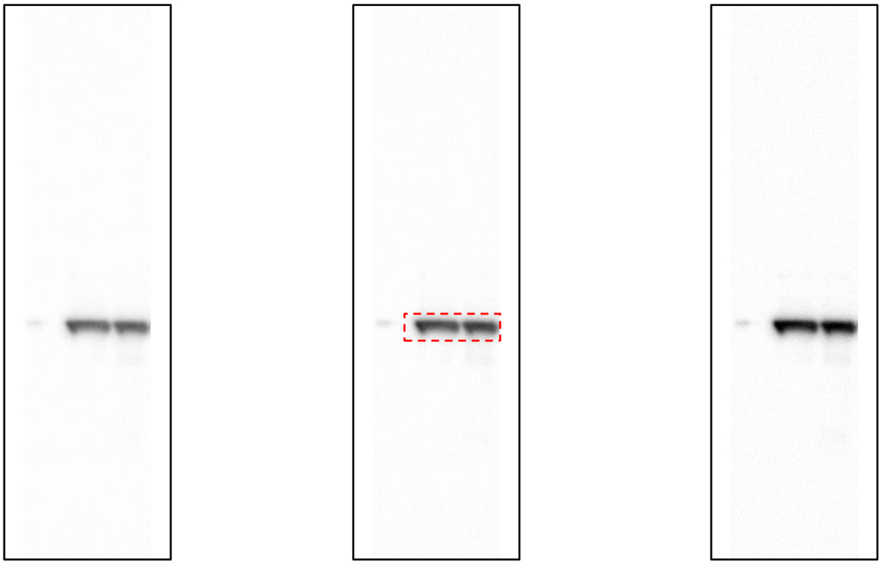

(1 second) (3 seconds) (5 seconds)

Supplementary Figure S3

(Full length blots of Figure 1F)

(Figure 1F)

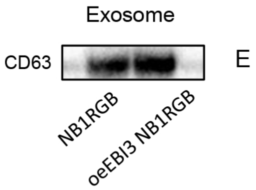

E

CD63

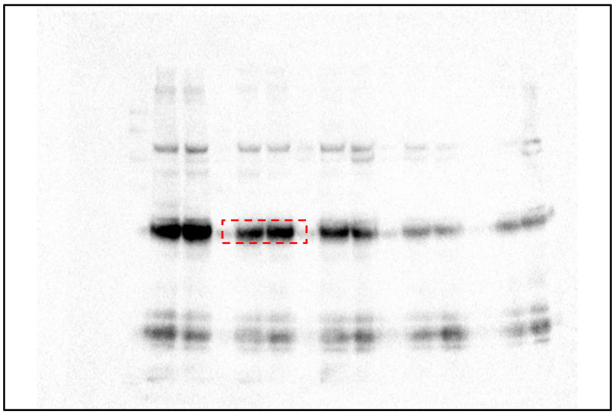

(3 seconds)

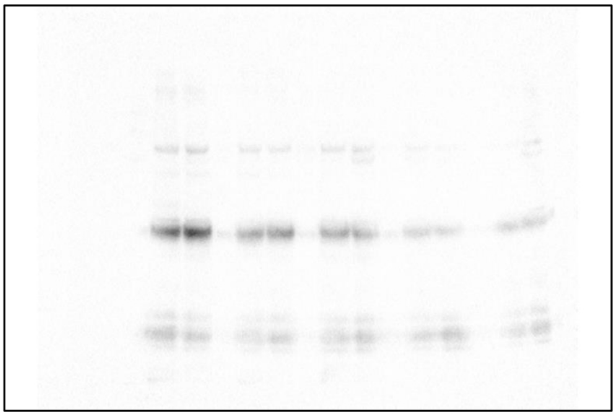

(1 second)

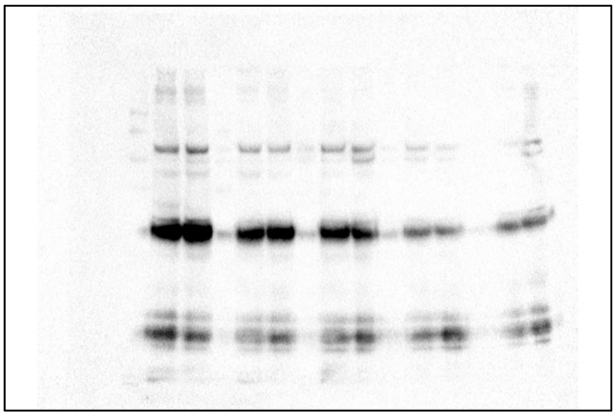

(5 seconds)

Supplementary Figure S3

(Full length blots of Figure 1F)

(Figure 1F)

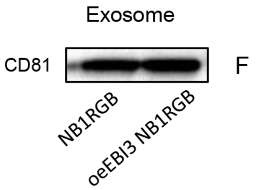

F

CD81

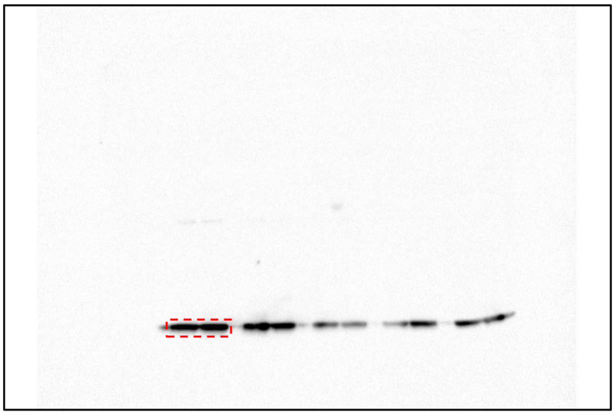

(3 seconds)

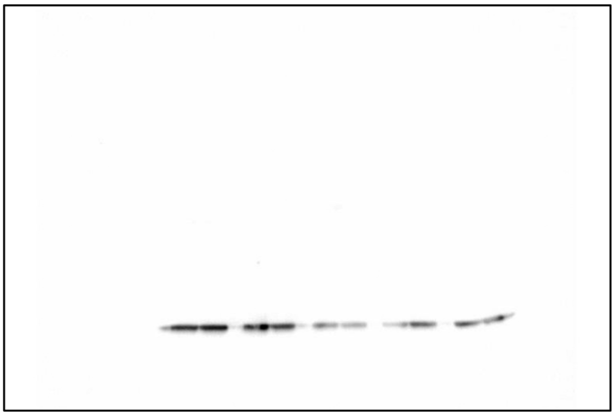

(1 second)

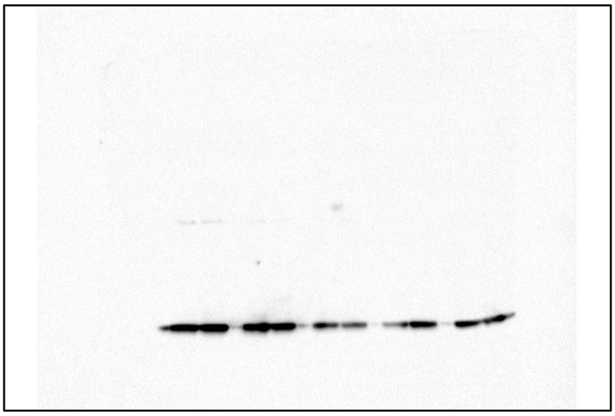

(5 seconds)

Supplementary Figure S4

(Fluorescent immunostaining image of Figure 2B)

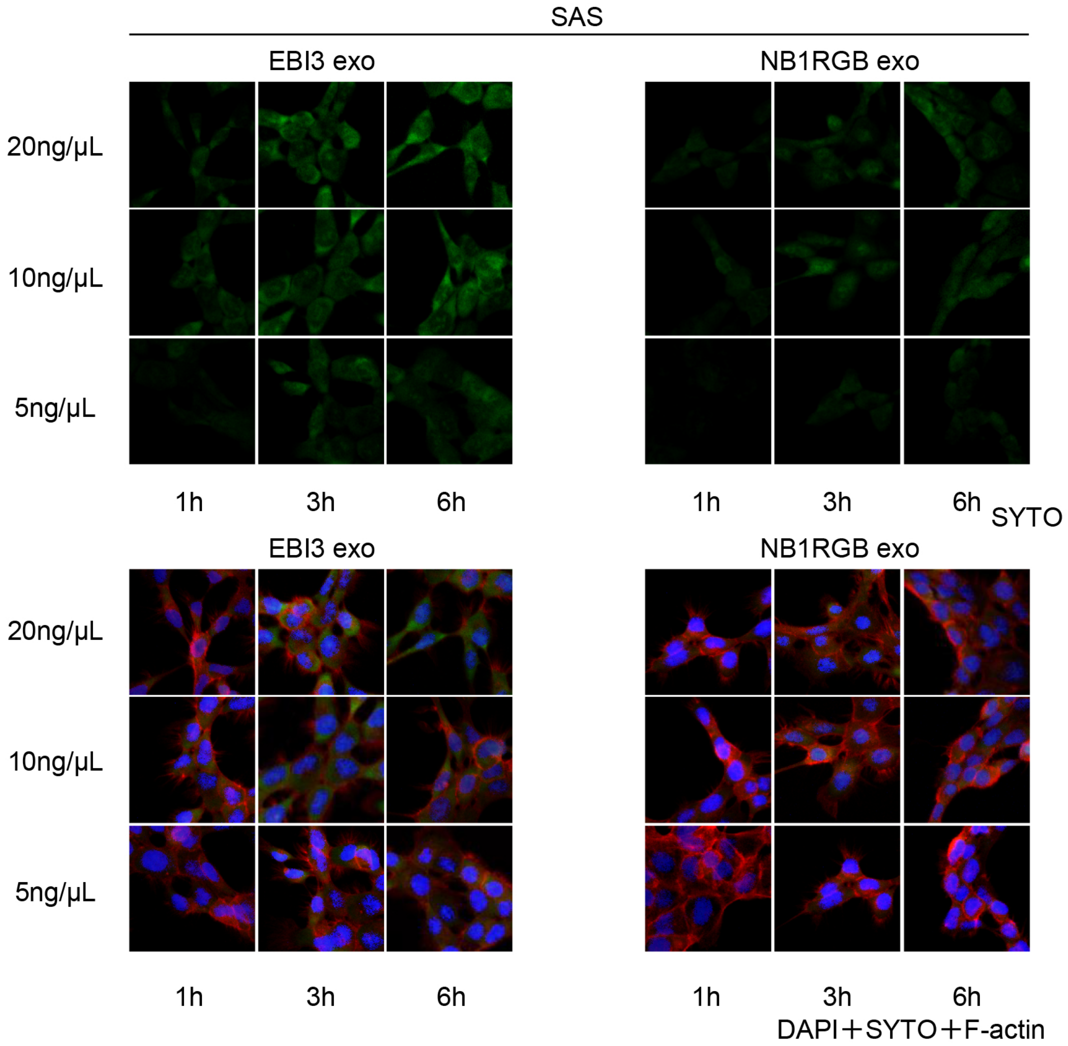

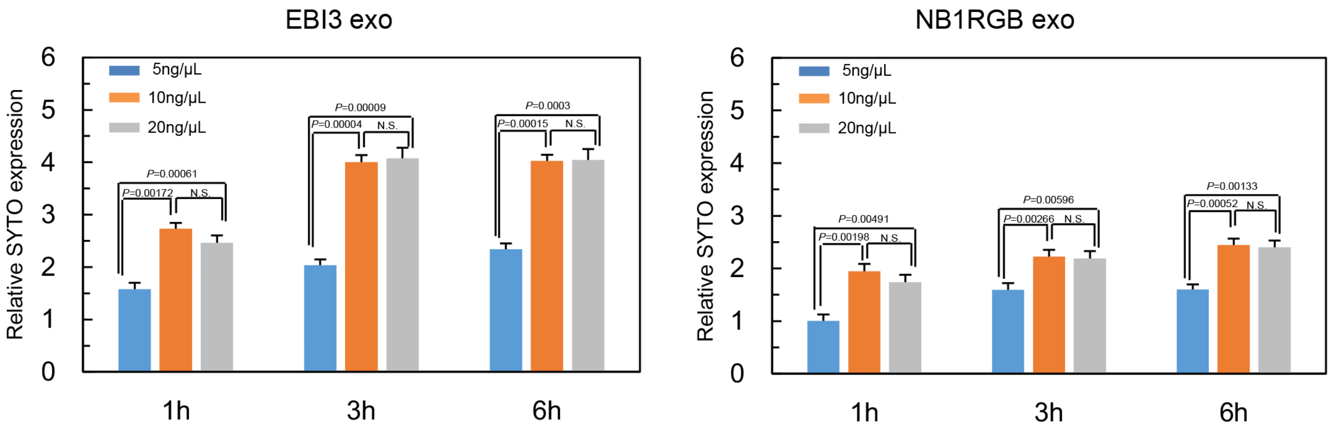

Supplementary Figure S5

(Full length blots of Figure 3B)

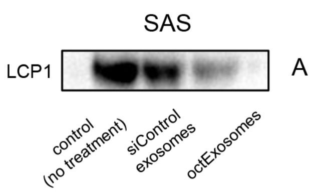

A

LCP1

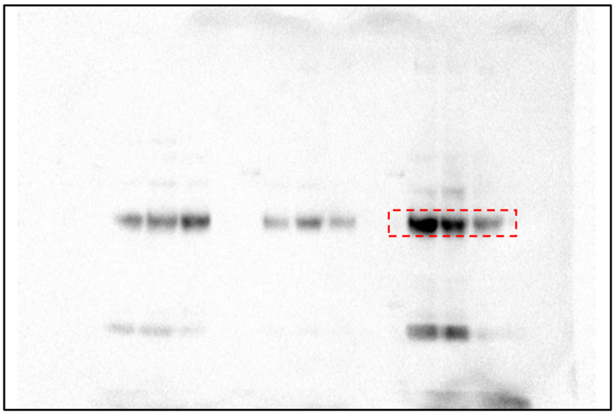

(3 seconds)

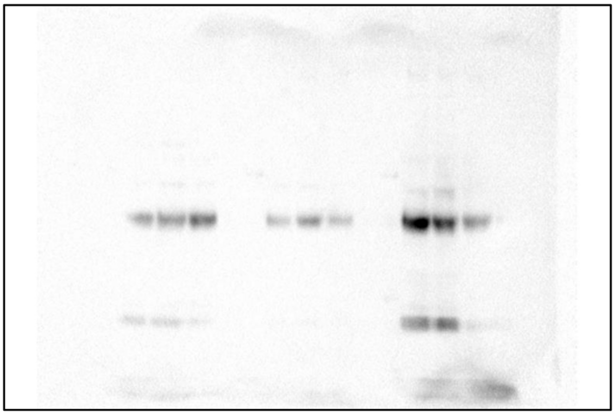

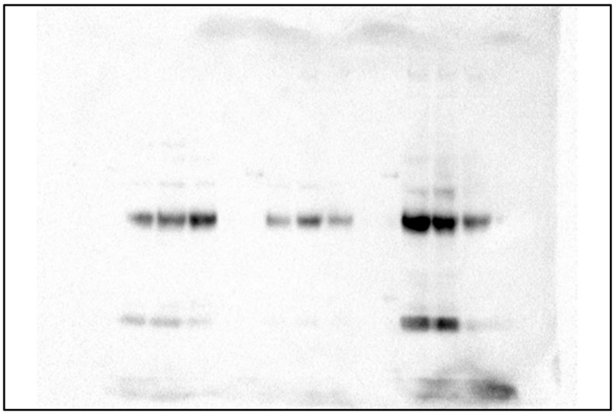

(1 second)

(5 seconds)

Supplementary Figure S5

(Full length blots of Figure 3B)

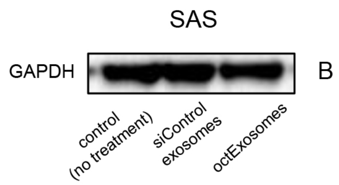

B

GAPDH

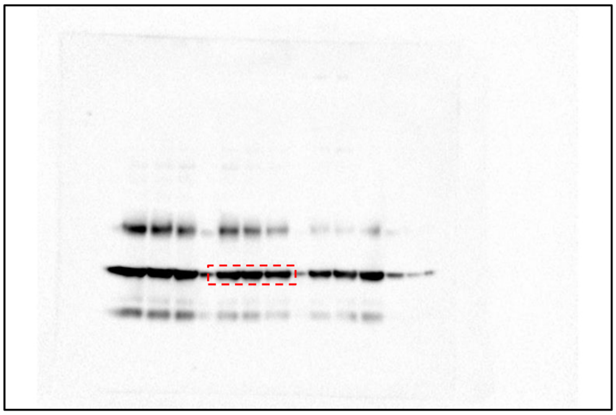

(3 seconds)

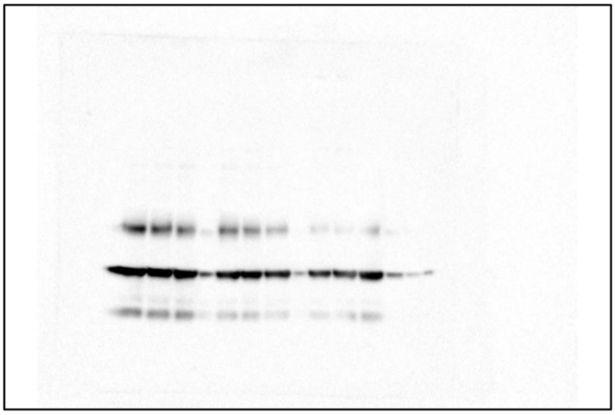

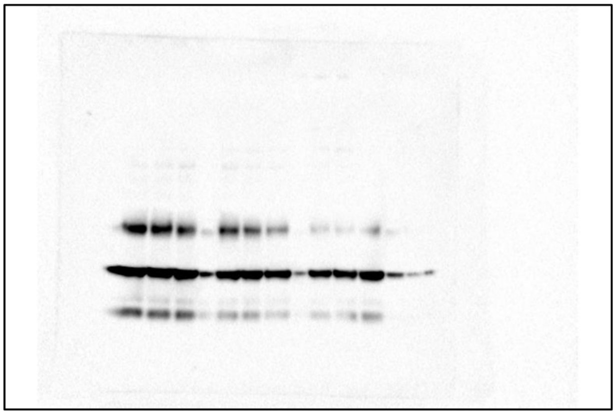

(1 second)

(5 seconds)

Supplementary Figure S5

(Full length blots of Figure 3B)

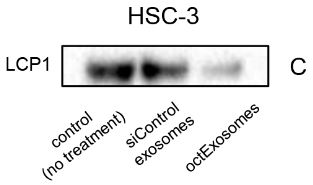

C

LCP1

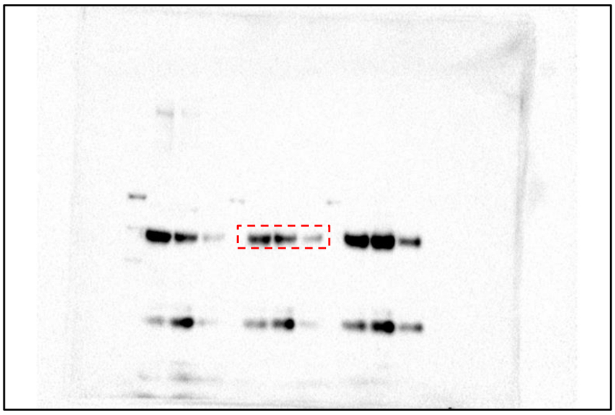

(3 seconds)

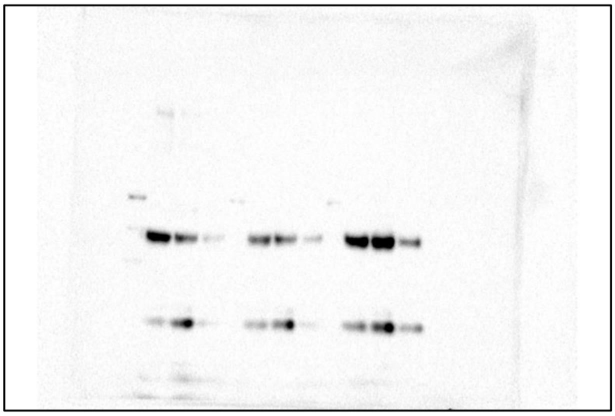

(1 second)

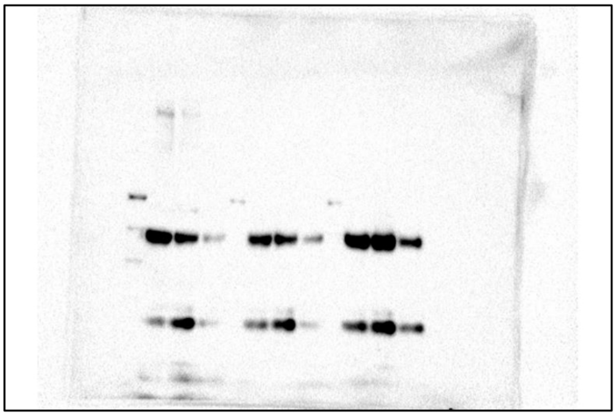

(5 seconds)

Supplementary Figure S5

(Full length blots of Figure 3B)

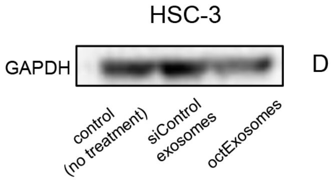

D

GAPDH

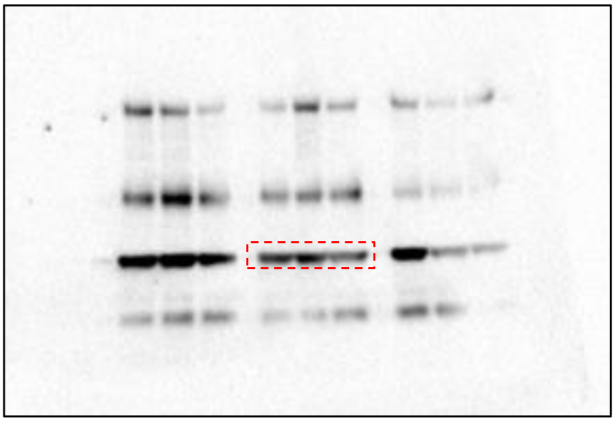

(3 seconds)

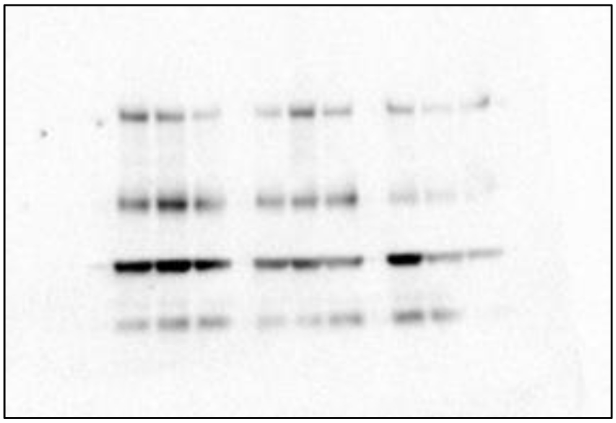

(1 second)

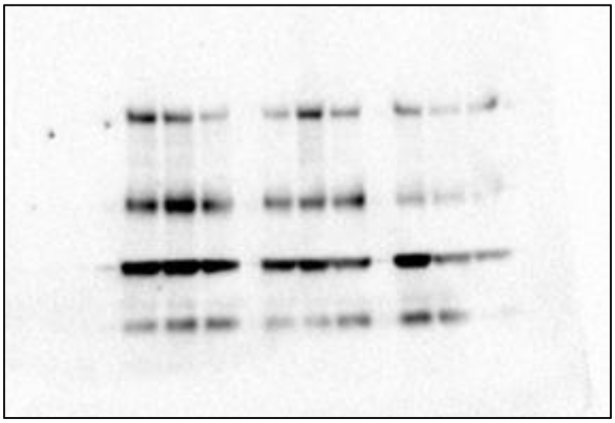

(5 seconds)

Supplementary Figure S6

(Full length blots of Figure 6B)

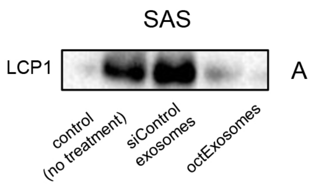

A

LCP1

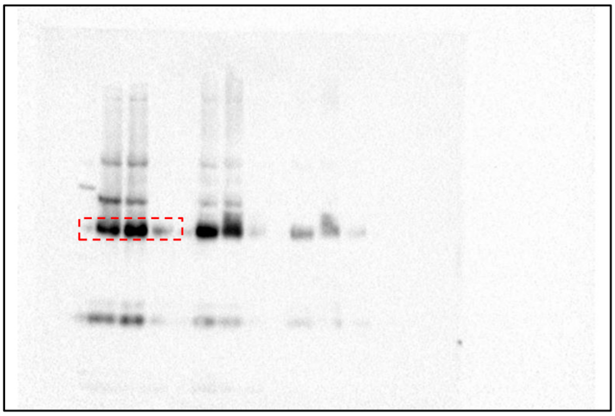

(3 seconds)

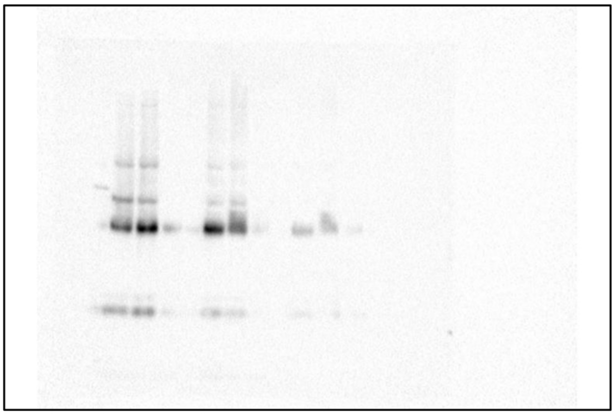

(1 second)

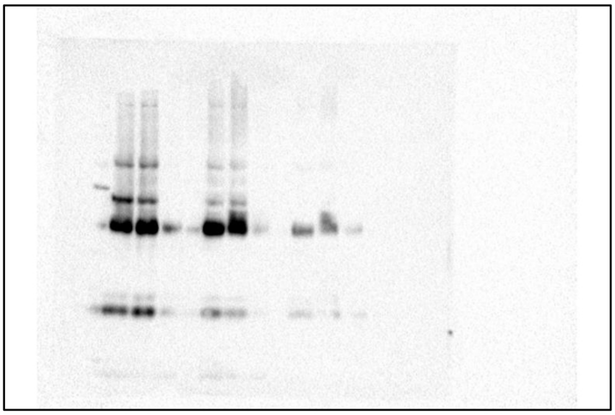

(5 seconds)

Supplementary Figure S6

(Full length blots of Figure 6B)

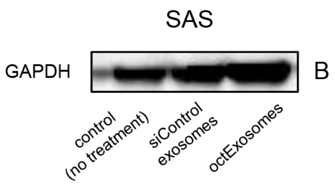

B

GAPDH

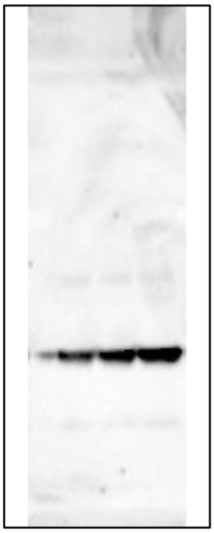

(1 second)

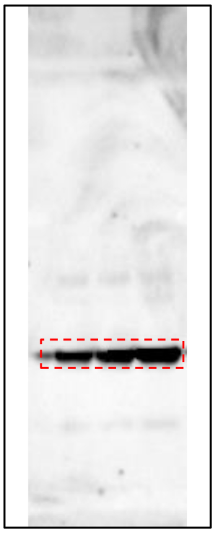

(3 seconds)

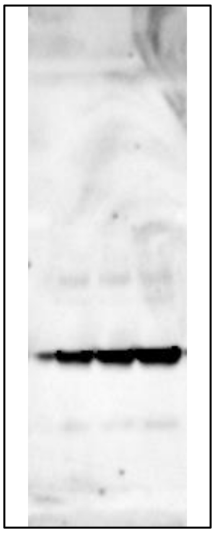

(5 seconds)

Supplementary Figure S6

(Full length blots of Figure 6B)

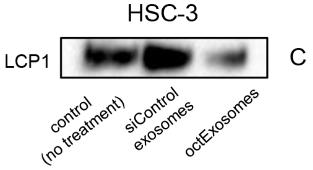

C

LCP1

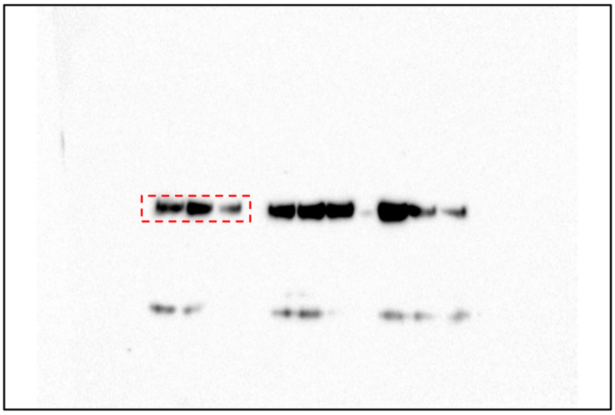

(3 seconds)

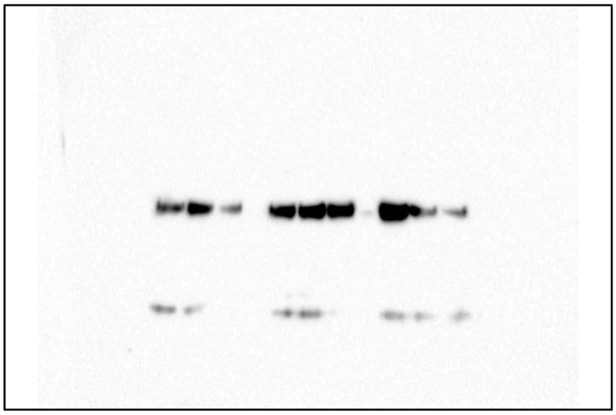

(1 second)

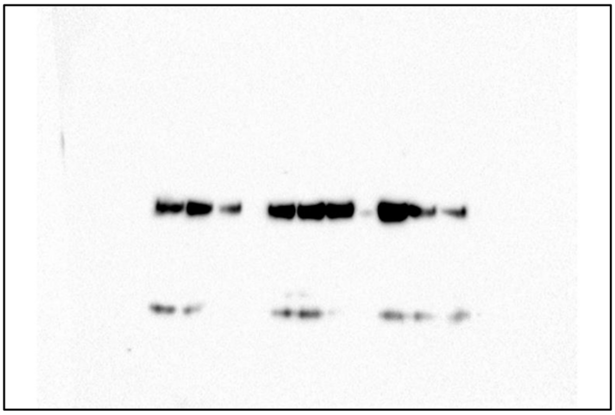

(5 seconds)

Supplementary Figure S6

(Full length blots of Figure 6B)

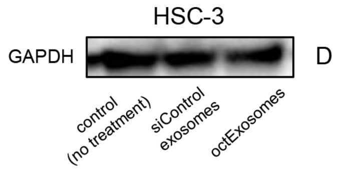

D

GAPDH

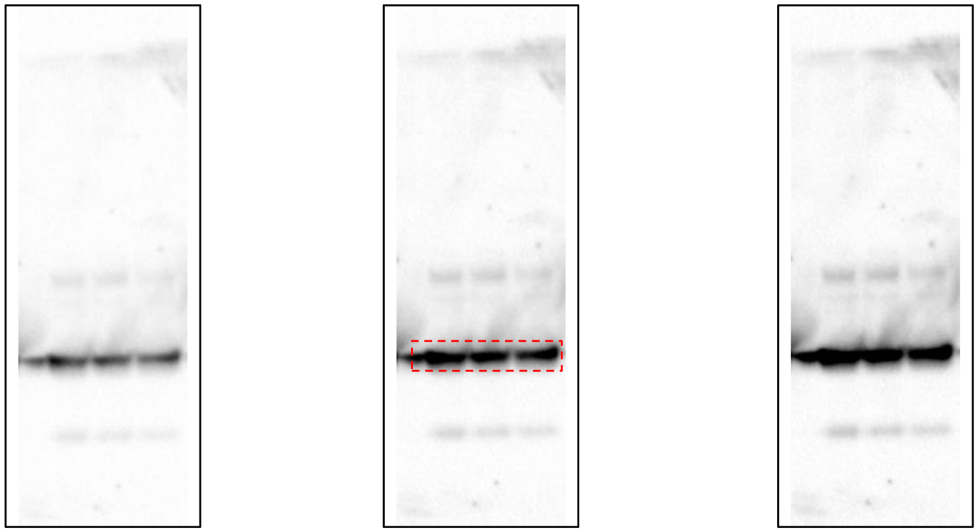

(1 second)

(3 seconds)

(5 seconds)
